# Epitope selection overcomes antigen shedding to enable effective CAR-M therapy in solid tumors

**DOI:** 10.1016/j.omton.2026.201354

**Published:** 2026-09-30

**Authors:** Wenlong Liu, Xiufen Liu, Seraphim Kozlov, Qi Zhou, Masanori Onda, Nadja Wagner, Ira Pastan

**Affiliations:** 1 Laboratory of Molecular Biology, National Cancer Institute, National Institutes of Health, Bethesda, MD 20892, USA; 2 Evotec International GmbH, Marie-Curie-Str. 7, 37079 Goettingen, Germany

**Keywords:** immunotherapy, CAR-macrophage, pancreatic cancer, colorectal cancer, antigen shedding

## Abstract

Chimeric antigen receptor-macrophages (CAR-Ms) represent a promising platform for solid tumors, but barriers limiting durable responses remain incompletely defined. Antigen shedding may be a key barrier because, unlike T cells, macrophages require sustained antigen engagement for effective phagocytosis. However, whether shed antigen directly impairs CAR-M function has not been established. Using mesothelin (MSLN) as a model target, we generated CAR-Ms targeting either an N-terminal epitope (SS1) or a membrane-proximal, non-shed epitope (15B6). Shed MSLN markedly impaired the phagocytic and cytotoxic activity of SS1 CAR-Ms, establishing antigen shedding as a direct functional barrier. In contrast, 15B6 CAR-Ms remained largely resistant and showed stronger antigen-specific activity across multiple MSLN^+^ tumor cells, demonstrating that rational epitope selection can overcome this barrier. In immunocompetent, MSLN-tolerant transgenic mouse models of colorectal and pancreatic cancer, 15B6 CAR-Ms demonstrated superior anti-tumor activity. In colorectal tumor-bearing mice, a single infusion of 15B6 CAR-Ms significantly prolonged survival, with 35% achieving complete tumor regression. Rechallenge of cured mice with MSLN^−^ tumor cells resulted in complete tumor rejection, suggesting the development of durable systemic anti-tumor immunity. Together, these findings identify antigen shedding as a critical barrier and support epitope selection as an important design strategy for CAR-M therapies targeting shed tumor antigens.

## Graphical abstract

**Figure:**
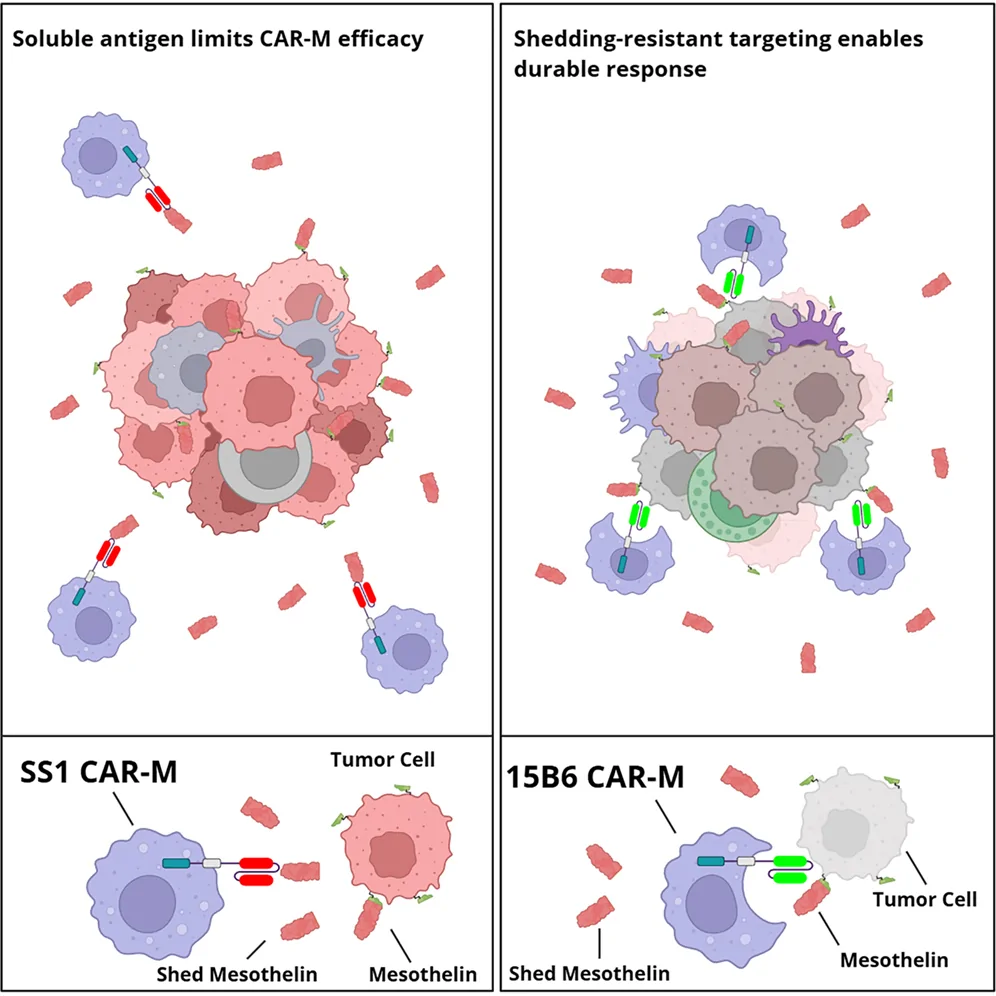

## Teaser

Liu and colleagues identify antigen shedding as a key barrier to CAR-macrophage therapy. By targeting a non-shed epitope of tumor antigen, they overcome this limitation, achieving tumor clearance and durable systemic immunity in immunocompetent transgenic mice. The work established epitope selection as a critical design principle for CAR-M therapies.

## Introduction

Macrophages play central roles in anti-tumor immunity through their capacity for tumor infiltration, phagocytosis, cytokine production, and antigen presentation. These properties have motivated the development of chimeric antigen receptor macrophages (CAR-Ms) as a therapeutic platform for solid tumors, where CAR-T cell therapies have shown limited efficacy due to poor trafficking, immunosuppressive tumor microenvironments (TMEs), and antigen heterogeneity.^1^^,^^2^ Preclinical studies have demonstrated that CAR-Ms can directly eliminate tumor cells and modulate the tumor immune landscape, supporting their potential as an alternative approach to CAR-T-based therapies.^3^^,^^4^

Mesothelin (MSLN) is a membrane glycoprotein overexpressed in a wide range of solid malignancies, including pancreatic and colorectal cancers,^5^ while exhibiting restricted expression in essential normal tissues.^6^ This expression pattern has made MSLN an attractive target for multiple immunotherapies including CAR-T, CAR-NK, and CAR-M approaches.^3^^,^^7^^,^^8^^,^^9^^,^^10^^,^^11^ However, a major challenge associated with MSLN targeting is protease-mediated shedding of its extracellular domain, resulting in high concentrations of soluble MSLN within the TME.^12^^,^^13^^,^^14^ In CAR-T cell therapies, soluble MSLN has been shown to interfere with receptor engagement and contribute to therapeutic resistance.^9^^,^^10^

This concern may be even more relevant in the context of CAR-M therapy. Unlike T cells, which can be activated through transient receptor-ligand interactions, macrophages require sustained target engagement and iterative receptor triggering throughout phagocytosis.^15^ Chronic exposure to soluble tumor antigens in the TME may therefore represent a significant barrier to CAR-M therapies in solid tumors. Consistent with this possibility, prior studies of MSLN-targeted CAR-Ms have reported limited or transient anti-tumor efficacy *in vivo*, despite effective CAR expression and tumor infiltration.^3^^,^^4^ However, whether antigen shedding constitutes a direct cause of CAR-M dysfunction remains unknown.

To investigate how human MSLN (hMSLN) shedding influences the activity of hMSLN-targeted CAR-Ms, immunocompetent models that tolerate hMSLN-expressing tumor cells and preserve macrophage-host immune interactions are required. This is particularly important given the central role of macrophages in coordinating innate immune functions and extensively engaging the host adaptive immune system. Although early studies established the feasibility of CAR-engineered macrophages across multiple targets,^3^^,^^4^^,^^16^^,^^17^^,^^18^ these approaches are predominantly performed in immunodeficient mouse models with a primary focus on phagocytic effector function, limiting assessment of their activity within intact immune systems.

Here, we address these gaps by engineering CAR-Ms expressing single-chain variable fragments (scFvs) derived from monoclonal antibodies that bind a membrane-proximal, non-shed epitope of hMSLN, thereby circumventing inhibition by soluble antigen. We evaluate this strategy using two complementary immunocompetent transgenic mouse models of pancreatic and colorectal cancer that are tolerant to hMSLN, enabling direct assessment of CAR-M function in the context of an intact host immune system. Notably, treatment with 15B6 CAR-Ms led to complete tumor regression in 35% of the treated mice and was associated with the development of systemic anti-tumor immunity capable of rejecting both hMSLN^+^ and hMSLN^−^ tumors. Together, our study identifies antigen shedding as a critical barrier to CAR-M efficacy in solid tumors and demonstrates that rational epitope selection can overcome this limitation, resulting in sustained anti-tumor activity and durable immune responses.

## Results

### Generation and functional characterization of 15B6 CAR-Ms

Murine bone marrow-derived cells were transduced during early myeloid differentiation in the presence of RetroNectin, prior to full macrophage maturation, followed by continued culture in M-CSF-containing medium to generate terminally differentiated CAR-expressing macrophages (Figure S1A). Cells were maintained in non-tissue culture-treated plates throughout the transduction and expansion process. All CAR constructs shared identical murine CD8 hinge and transmembrane domains and a murine CD3ζ intracellular activation domain, differing only in their extracellular targeting scFvs (Figure 1A). Specifically, 15B6 CAR-Ms incorporated a 15B6 scFv targeting a membrane-proximal, non-shed epitope of hMSLN, SS1 CAR-Ms contained the conventional SS1 scFv recognizing the N-terminal domain of hMSLN, and FMC63 CAR-Ms served as a non-targeting control directed against human CD19.

**Figure 1:**
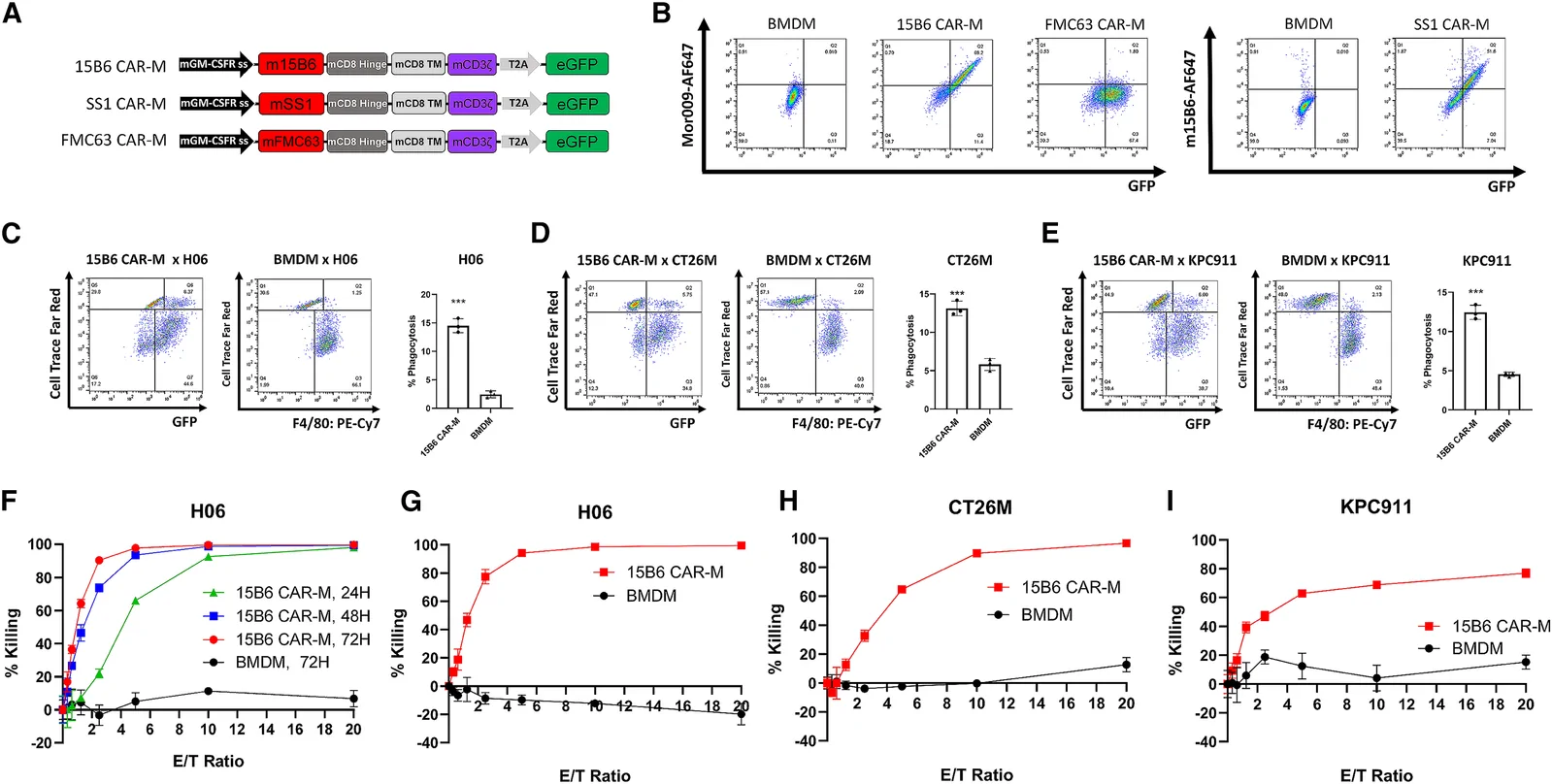
Engineered 15B6 CAR-Ms actively phagocytose and kill hMSLN^+^ tumor cells (A) Schematic diagram of CAR constructs. (B) 15B6 CAR-Ms and SS1 CAR-Ms bind to hMSLN, while FMC63 CAR-Ms do not. BMDMs from mice with a C57BL/6 background were transduced with lentivirus; successfully transduced CAR-Ms were GFP^+^. Cells were then incubated with hMSLN (296-599-His) on ice, followed by anti-hMSLN staining (Mor009 binds to the N terminus of hMSLN, whereas m15B6 binds to the C terminus of hMSLN). Transduced cells that bind to hMSLN were AF647^+^ GFP^+^. %Binding = (% AF647^+^ GFP^+^ cells/% GFP^+^ cells) × 100%. (C–E) 15B6 CAR-Ms phagocytosed hMSLN^+^ tumor cells (H06/CT26M/KPC911). H06, CT26M, or KPC911 cells were labeled with CellTrace FarRed, then co-cultured with 15B6 CAR-Ms or BMDMs for 4 h in 96-well low-adherent plates at E/T ratio = 1. %Phagocytosis = (M^+^ FarRed^+^ cells/M^+^ cells) × 100%. M represents effector cells (marked by either GFP, F4/80, or CD11b), depending on the experiment. (F) 15B6 CAR-Ms killed hMSLN^+^ H06 cells in a time-dependent manner. 15B6 CAR-Ms or BMDMs were co-cultured with Luc^+^ H06 cells on 96-well plates at different E/T ratios for 24, 48, and 72 h. (G–I) 15B6 CAR-Ms, rather than BMDMs, killed hMSLN^+^ H06/CT26M/KPC911 cells. 15B6 CAR-Ms or BMDMs were co-cultured with Luc^+^ H06/CT26M/KPC911 cells on 96-well plates at different E/T ratios for 48 h. Data are presented as mean ± SD. Statistical significance is indicated as follows: ∗*p* < 0.05, ∗∗*p* < 0.01, ∗∗∗*p* < 0.001, and ∗∗∗∗*p* < 0.0001; ns, not significant.

Three days after lentiviral transduction in the presence of M-CSF, GFP expression—encoded within the same lentiviral vector as the CAR construct—was used as a surrogate marker to assess viral transduction efficiency. At this stage, bone marrow-derived macrophages (BMDMs) from C57BL/6 background mice exhibited GFP positivity of 86.4% for 15B6 CAR-Ms, 73.3% for SS1 CAR-Ms, and 80.7% for FMC63 CAR-Ms, while BALB/c-derived BMDMs showed GFP positivity of 81.4%, 68.6%, and 77.9%, respectively (Figure S1B). To functionally validate scFv-mediated antigen binding, the interaction of CAR-Ms with soluble hMSLN was assessed. Both 15B6 CAR-Ms and SS1 CAR-Ms demonstrated robust hMSLN binding, with 85.9% and 88.0% of GFP^+^ cells exhibiting hMSLN binding, respectively, whereas the non-targeting FMC63 CAR-Ms showed no detectable binding (Figure 1B). Comparable binding activity was observed in BALB/c-derived CAR-Ms (Figure S1B), confirming consistent scFv-mediated hMSLN recognition across both mouse backgrounds.

Phagocytosis assays were performed to evaluate the activity of 15B6 CAR-Ms against various hMSLN-expressing tumor cell lines. As shown in Figures 1C–1E, GFP^+^ 15B6 CAR-Ms exhibited significantly enhanced phagocytic activity compared with untransduced BMDMs, with increases of 494% for H06 (*p* < 0.001, *n* = 3), 126% for CT26M (*p* < 0.001, *n* = 3), and 174% for KPC911 (*p* < 0.001, *n* = 3), respectively.

To assess cytotoxic activity over time, luciferase-expressing (Luc^+^) H06 cells were co-cultured with 15B6 CAR-Ms in a time-course killing assay. Although the transient phagocytosis measured at an early point was relatively modest (Figures 1C–1E), CAR-M-mediated tumor cell elimination accumulated over time at each specific effector-to-target (E/T) ratio, reaching maximal levels within 48 h at E/T ratios ≥ 10 (Figure 1F). After 48 h of co-culture (E/T ratio = 20), 15B6 CAR-Ms achieved over 95% killing of H06 (IC_50_ = 0.978) and CT26M (IC_50_ = 3.876) cells (Figures 1G and 1H). For KPC911 cells, 15B6 CAR-Ms reached a maximal killing rate of 77% (IC_50_ = 1.349) (Figure 1I). To determine whether incorporation of additional signaling domains could further enhance cytotoxic activity, we generated modified 15B6-based CAR-M constructs incorporating 4-1BB, CD86, or TLR4 signaling elements and evaluated them against Luc^+^ H06 cells. The results show that none of these modifications improved 15B6 CAR-M’s cytotoxicity against H06 cells (Figures S2A and S2B). Interestingly, CAR-Ms containing co-stimulatory domains exhibited lower percentages of hMSLN-binding cells (38.6%–83.4%) than the original 15B6 CAR-Ms (88.7%) (Figure S2C), suggesting that reduced surface expression of functional scFv may partially contribute to their decreased activity.

Notably, successfully transduced 15B6 CAR-Ms could be expanded during early passages, with total cell numbers increasing more than 10-fold over an 18- to 19-day culture period (Figure S3). Collectively, these results demonstrate efficient generation of functional CAR-Ms from murine BMDMs. The resulting 15B6 CAR-Ms exhibited robust phagocytic and cytotoxic activity against multiple hMSLN-expressing tumor cells and retained the capacity for controlled *ex vivo* expansion.

### 15B6 CAR-Ms mediate antigen-specific tumor killing and activation upon hMSLN engagement

To assess antigen specificity, we compared the activity of 15B6 CAR-Ms against hMSLN^+^ and hMSLN^−^ tumor cells. 15B6 CAR-Ms selectively phagocytosed hMSLN^+^ H06 cells but not their parental hMSLN^−^ PDA223481 cells. In contrast, phagocytic activity against H06 cells remained at basal levels (<5%) in untransduced BMDMs and non-targeting FMC63 CAR-M controls (Figures 2A and 2B).

**Figure 2:**
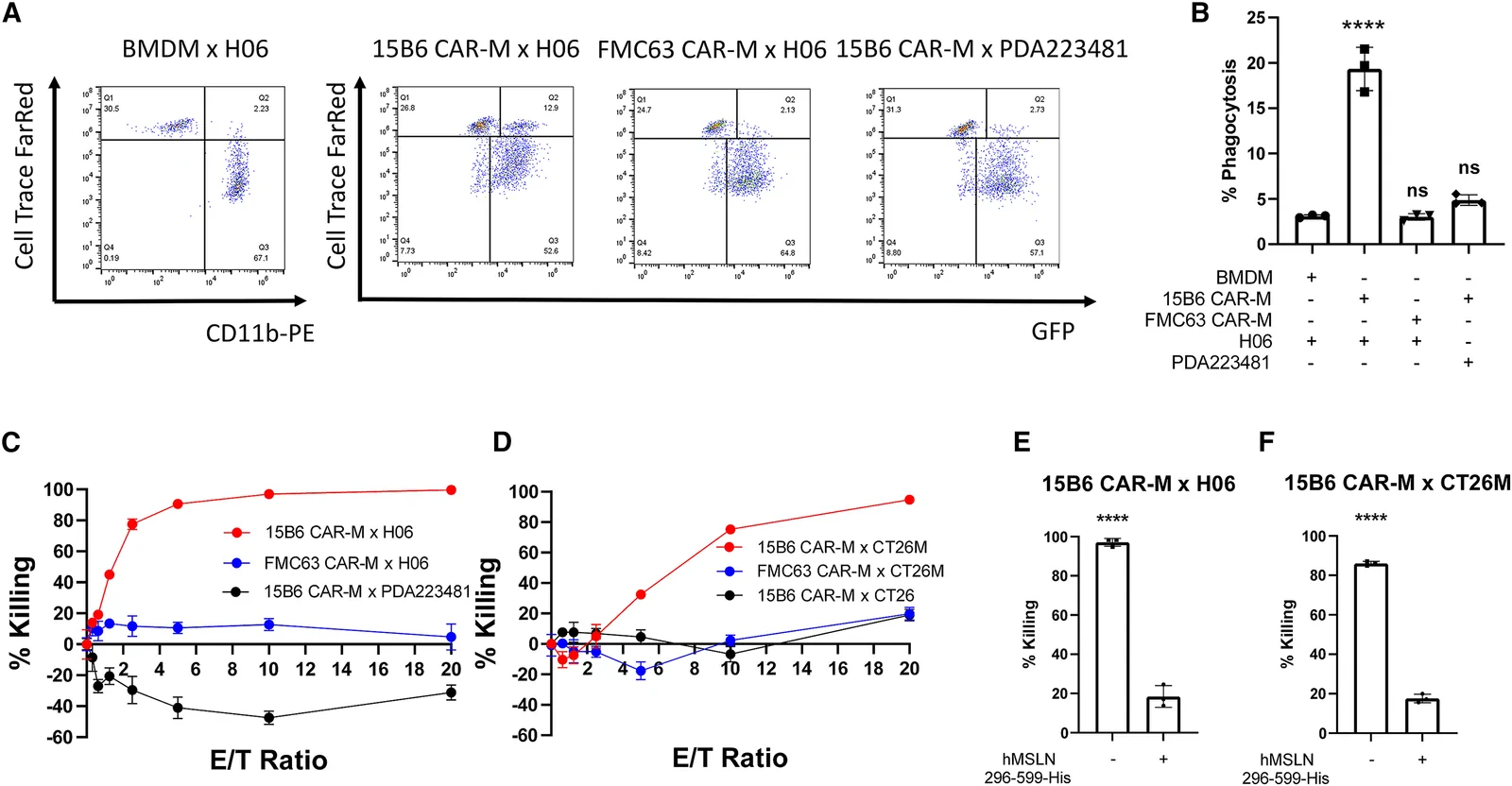
The 15B6 CAR-Ms exhibited hMSLN-specific phagocytic and killing activities (A and B) 15B6 CAR-Ms exhibited hMSLN-specific phagocytosis activity. hMSLN^+^ H06 and its parental hMSLN^−^ cells (PDA223481) were labeled with CellTrace FarRed, then co-cultured with 15B6 CAR-Ms, FMC63 CAR-Ms, or BMDMs for 4 h in 96-well low-adherent plates at E/T ratio = 1. %Phagocytosis = (M^+^ FarRed^+^ cells/M^+^ cells) × 100%. M represents effector cells (marked by either GFP, F4/80, or CD11b), depending on the experiment. (C) 15B6 CAR-Ms exhibited hMSLN-specific cytotoxicity toward H06 cells. 15B6 CAR-Ms or FMC63 CAR-Ms were co-cultured with Luc^+^ H06 cells or PDA223481 cells on 96-well plates at different E/T ratios for 48 h. (D) 15B6 CAR-Ms exhibited hMSLN-specific cytotoxicity on CT26M cells. 15B6 CAR-Ms or FMC63 CAR-Ms were co-cultured with Luc^+^ CT26M cells or CT26 cells on 96-well plates at different E/T ratios for 48 h. (E) The killing activity of 15B6 CAR-Ms against H06 cells was blocked by full-length hMSLN. 15B6 CAR-Ms were co-cultured with Luc^+^ H06 cells with or without 50 μg/mL hMSLN 296-599-His at E/T ratio = 10 for 48 h. (F) The killing activity of 15B6 CAR-Ms against CT26M cells was blocked by full-length hMSLN. 15B6 CAR-Ms were co-cultured with Luc^+^ CT26M cells with or without 50 μg/mL hMSLN 296-599-His at E/T ratio = 20 for 48 h. Data are presented as mean ± SD. Statistical significance is indicated as follows: ∗*p* < 0.05, ∗∗*p* < 0.01, ∗∗∗*p* < 0.001, and ∗∗∗∗*p* < 0.0001; ns, not significant.

Consistently, 15B6 CAR-Ms induced dose-dependent killing of hMSLN^+^ H06 cells (Figure 2C) and hMSLN^+^ CT26M colorectal tumor cells (Figure 2D), whereas the non-targeting FMC63 CAR-Ms showed no significant cytotoxic activity. No detectable cytotoxicity was observed against hMSLN^−^ pancreatic (PDA223481) or colorectal (CT26) tumor cells, even at E/T ratios as high as 20 (Figures 2C and 2D). As shown previously, untransduced BMDMs exhibited minimal cytotoxicity under comparable conditions (Figures 1G and 1H). To further determine whether cytotoxic activity requires direct CAR-antigen engagement, full-length soluble hMSLN containing the 15B6 binding epitope (MSLN 296-599-His) was introduced, and it reduced killing of H06 (Figure 2E) and CT26M (Figure 2F) cells by 81% and 80%, respectively.

Together, these results demonstrate that 15B6 CAR-M-mediated phagocytosis and cytotoxicity are antigen dependent and require direct interaction between the hMSLN-specific CAR and hMSLN expressed on tumor cells. In the absence of antigen engagement, a relative increase in PDA223481 cell number under co-culture conditions was observed, whereas no such effect was detected in CT26 cells (Figures 2C and 2D).

Consistent with antigen-dependent activation, 15B6 CAR-Ms exhibited increased expression of the M1-associated markers CD80 and CD86 when co-cultured with hMSLN^+^ H06 cells compared to hMSLN^−^ PDA223481 cells (Figures 3A–3D). In contrast, non-transduced BMDMs did not show increased CD80 or CD86 expression under the same conditions. These findings suggest that direct CAR engagement induces M1-associated phenotypic changes in 15B6 CAR-Ms.

**Figure 3:**
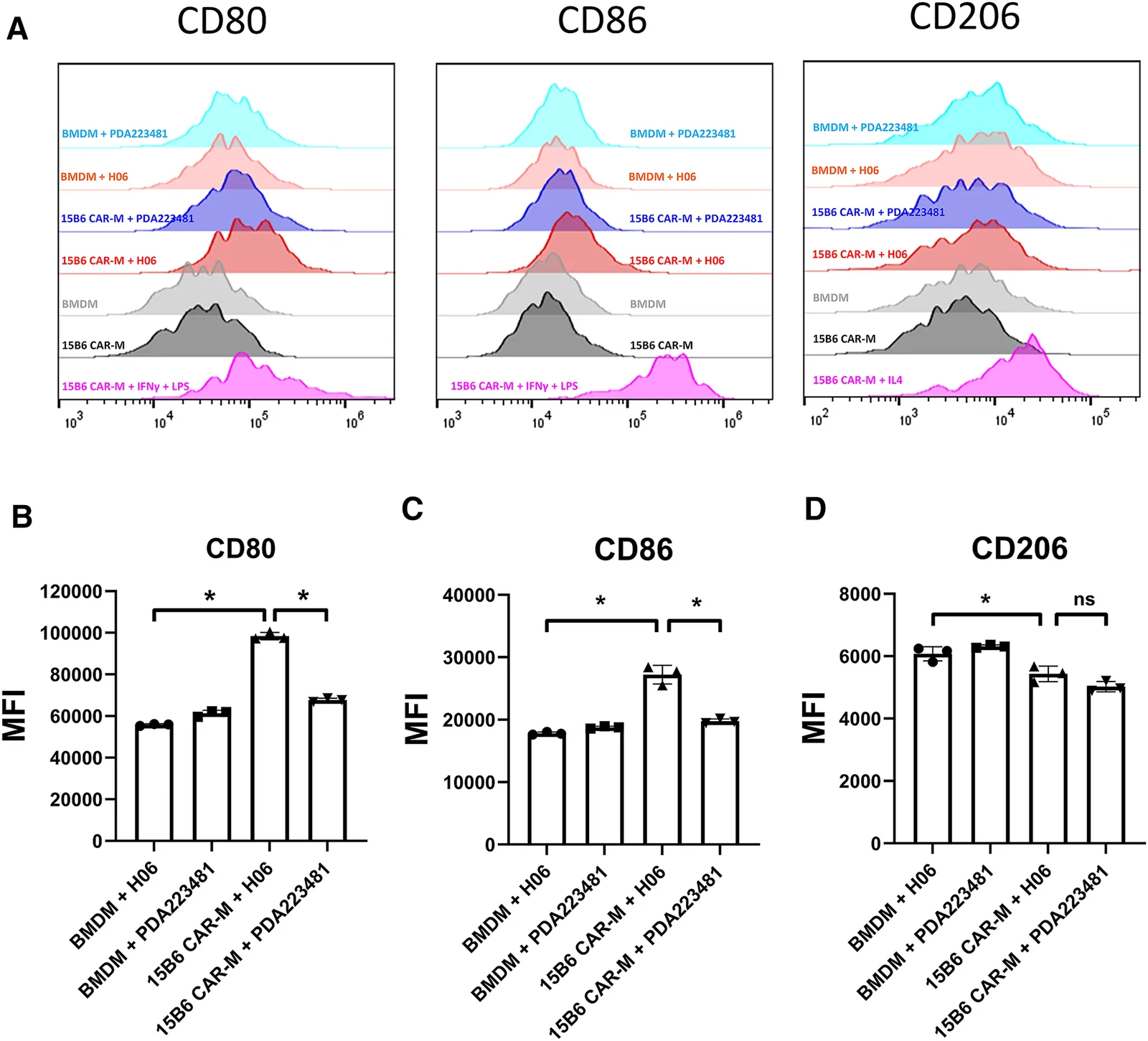
15B6 CAR-Ms expressed higher M1-associated markers when co-cultured with hMSLN^+^ H06 cells (A) 15B6 CAR-Ms or BMDMs were co-cultured with hMSLN^+^ H06 or hMSLN^−^ PDA223481 cells on 96-well plates at E/T ratio = 1 for 48 h. Macrophage (15B6 CAR-Ms: GFP^+^ F4/80^+^, BMDMs: F4/80^+^) polarization activities were compared by their geometric mean fluorescence intensity (MFI). (B–D) Reads from flow cytometry measurements (M1-associated: CD80 and CD86; M2-associated: CD206). Data are presented as mean ± SD. Statistical significance is indicated as follows: ∗*p* < 0.05, ∗∗*p* < 0.01, ∗∗∗*p* < 0.001, and ∗∗∗∗*p* < 0.0001; ns, not significant.

### 15B6 CAR-Ms exhibit enhanced anti-tumor activity compared with SS1 CAR-Ms by targeting a non-shed MSLN epitope

The anti-tumor activity of 15B6 CAR-Ms, which targets a membrane-proximal, non-shed epitope of hMSLN, was compared with that of conventional SS1 CAR-Ms, which recognizes an N-terminal epitope subject to proteolytic shedding. As shown in Figures 4A–4C, 15B6 CAR-Ms exhibited significantly higher phagocytic activity against pancreatic tumor H06 cells (148% increase, *p* < 0.0001, *n* = 3) and KPC911 cells (32% increase, *p* < 0.005, *n* = 3) compared with SS1 CAR-Ms, whereas only a modest difference was observed for colorectal tumor CT26M cells (15% increase, *p* = 0.08, *n* = 3).

**Figure 4:**
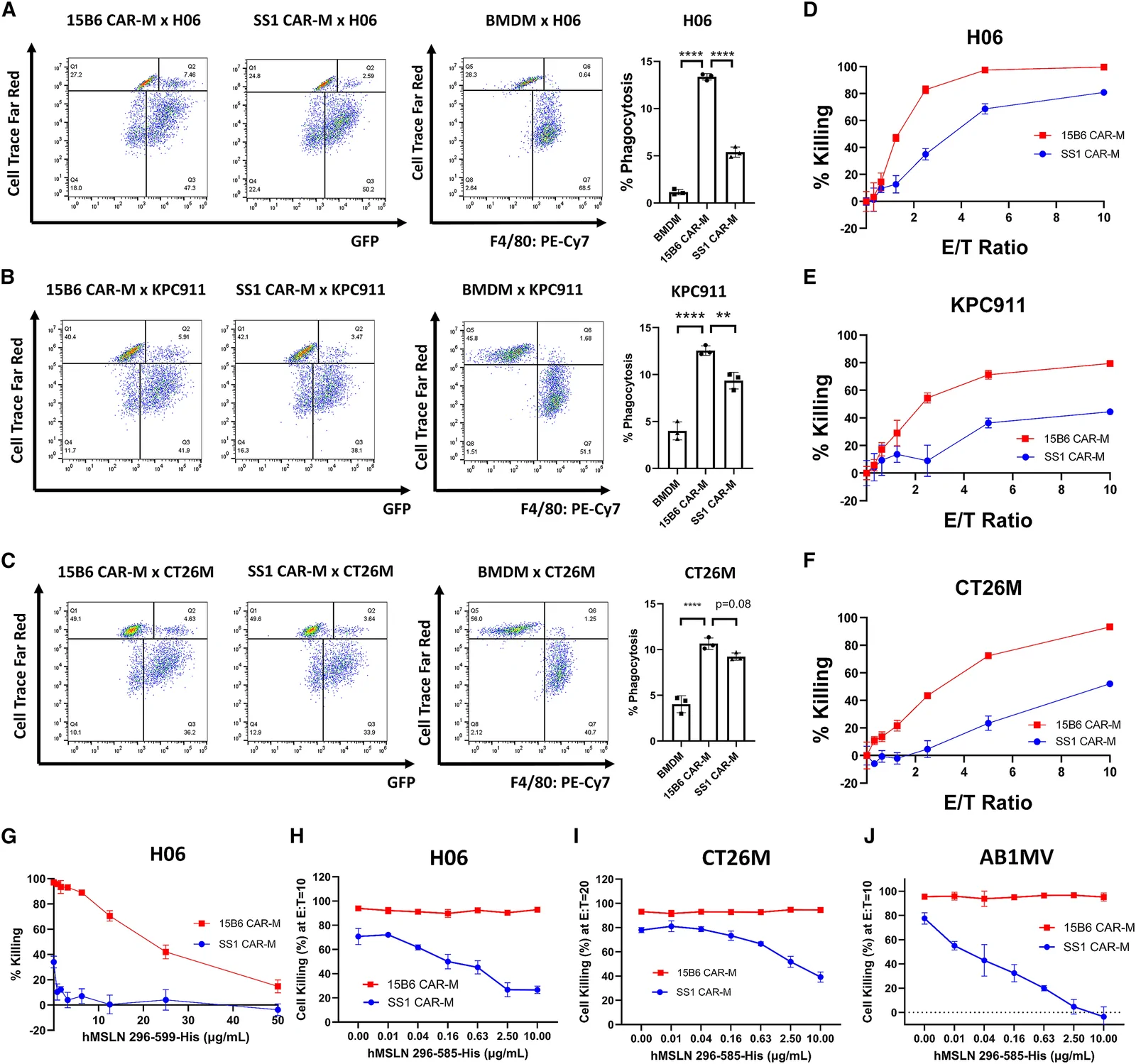
15B6 CAR-Ms exhibited greater phagocytosis, cytotoxicity, and resistance to shedding hMSLN than SS1 CAR-Ms (A–C) 15B6 CAR-Ms show better phagocytosis activity on multiple tumor cells than SS1 CAR-Ms. H06, KPC-911, or CT26M cells were labeled with CellTrace FarRed, then co-cultured with 15B6 CAR-Ms or SS1 CAR-Ms for 4 h in 96-well low-adherent plates at E/T ratio = 1. %Phagocytosis = (M^+^ FarRed^+^ cells/M^+^ cells) × 100%. M represents effector cells (marked by either GFP, F4/80, or CD11b), depending on the experiment. (D–F) 15B6 CAR-Ms show better killing activity on multiple tumor cells than SS1 CAR-Ms. 15B6 CAR-Ms or SS1 CAR-Ms were co-cultured with Luc^+^ H06/KPC-911/CT26M cells on 96-well plates at different E/T ratios for 48 h. (G) Full-length hMSLN inhibited the killing activity of both 15B6 CAR-Ms and SS1 CAR-Ms. (H–J) Shed hMSLN inhibited the killing activity of SS1 CAR-Ms while not influencing the killing activity of 15B6 CAR-Ms. 15B6 CAR-Ms or SS1 CAR-Ms were co-cultured with Luc^+^ H06 (E/T ratio = 10), CT26M (E/T ratio = 20), and AB1MV (E/T ratio = 10) cells on 96-well plates with either hMSLN 296-585-His (shed hMSLN) or hMSLN 296-599-His (full-length hMSLN) at the concentrations indicated in the corresponding panels. The killing efficiency was measured at 48 h. Data are presented as mean ± SD. Statistical significance is indicated as follows: ∗*p* < 0.05, ∗∗*p* < 0.01, ∗∗∗*p* < 0.001, and ∗∗∗∗*p* < 0.0001; ns, not significant.

Consistently, 15B6 CAR-Ms showed enhanced cytotoxicity compared to SS1 CAR-Ms across multiple hMSLN^+^ tumor cell lines, including H06, KPC911, CT26M, and mesothelioma (AB1MV) cells (Figures 4D–4F and S4).

To determine whether soluble hMSLN differentially affects CAR-M activity depending on epitope location, recombinant full-length hMSLN (MSLN 296-599-His), as well as physiologically shed hMSLN (represented by MSLN 296-585-His), which lacks the membrane-proximal 15B6 epitope, was added to co-culture assays.

As expected, the full-length construct inhibited the cytotoxicity of both 15B6 CAR-Ms and SS1 CAR-Ms (Figure 4G), indicating that CAR-hMSLN interaction is required for antigen-specific tumor killing by both constructs. Most importantly, in the presence of shed hMSLN (MSLN 296-585-His), the cytotoxicity of SS1 CAR-Ms was significantly reduced against H06 (62% inhibition, *p* < 0.001, *n* = 3), CT26M (50% inhibition, *p* = 0.0001, *n* = 3), and AB1MV (complete inhibition, *p* = 0.0001, *n* = 3) cells. In contrast, the cytotoxic activity of 15B6 CAR-Ms remained largely unaffected under the same conditions (Figures 4H–4J).

### 15B6 CAR-Ms exhibit enhanced anti-tumor activity in immunocompetent hMSLN-tolerant models

In addition to direct cytotoxicity, macrophages actively interact with the adaptive immune system by functioning as antigen-presenting cells (APCs) and producing cytokines that shape immune responses. To evaluate the anti-tumor activity of 15B6 CAR-Ms in immunocompetent settings, we utilized two transgenic mouse models tolerant to hMSLN: Thyroid Peroxidase (TPO)-MSLN mice and hMSLN mice. TPO-MSLN mice express hMSLN exclusively on the thyroid,^7^^,^^19^ whereas hMSLN mice express hMSLN in tissues corresponding to endogenous murine Msln expression.^20^ Both models possess intact immune systems and do not reject hMSLN^+^ tumor cells.

In the colorectal tumor TPO-MSLN model, 15B6 CAR-Ms significantly improved overall survival compared with vehicle (Veh) control (Figures 5A–5C). 6 of 17 mice achieved tumor complete regression (CR) in the 15B6 CAR-M group, whereas all vehicle-treated mice died within 35 days post-tumor inoculation (Figures 5B and 5C). In contrast, in immunocompromised NOD.Cg-*Prkdc*^scid^ *Il12rg*^tm1Wjl^/SzJ (NSG) mice bearing CT26M tumors, 15B6 CAR-M treatment extended survival but did not result in CR (Figures S5A–S5C).

**Figure 5:**
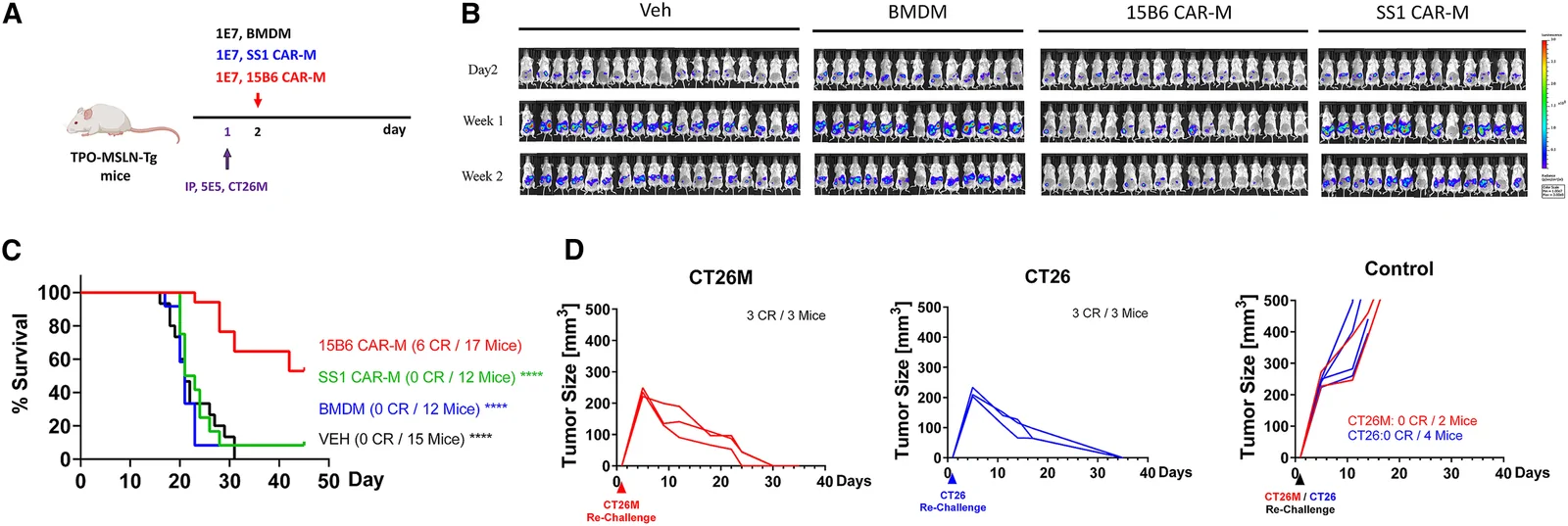
15B6 CAR-Ms eradicated colorectal tumor in TPO-MSLN mouse model and induced anti-tumor immunity (A) TPO-MSLN mice were inoculated (i.p.) with 0.5 × 10^6^ CT26M cells on day 1 and treated (i.p.) with 1 × 10^7^ 15B6 CAR-Ms, SS1 CAR-Ms, BMDMs, or DMEM (Veh) 24 h post-tumor inoculation. (B) IVIS images of tumors in mice received different treatments. (C) The survival curve of mice received different treatments. (D) At least 45 days after tumor inoculation, the CR mice were subcutaneously rechallenged with either 2 × 10^6^ hMSLN^+^ CT26M cells (*n* = 3) or hMSLN^−^ CT26 cells (*n* = 3), and tumor size was measured. Control groups were TPO-MSLN mice (*n* = 2) inoculated with CT26M cells or BALB/c mice (*n* = 4) inoculated with CT26 cells.

To further assess anti-tumor activity in an independent immunocompetent model, hMSLN transgenic mice were inoculated with hMSLN^+^ murine pancreatic cancer cells (H06). In this setting, 15B6 CAR-M treatment significantly reduced tumor bioluminescence signals (Figures S6A and S6B) and tumor weight (Figure S6C) compared with vehicle controls. For long-term follow-up, three mice per group were monitored beyond the endpoint used for tumor weight analysis. All three monitored mice in the 15B6 CAR-M group achieved complete tumor regression (Figure S6A).

Consistent with the *in vitro* findings showing that 15B6 CAR-Ms exhibit enhanced cytotoxic activity and are resistant to inhibition by shed hMSLN (Figure 4), 15B6 CAR-Ms significantly prolonged survival in TPO-MSLN mice bearing CT26M tumors compared with both SS1 CAR-Ms (*p* < 0.0001) and BMDMs (*p* < 0.0001) (Figures 5B and 5C). In contrast, SS1 CAR-Ms demonstrated minimal survival benefit relative to BMDM controls (*p* = 0.31), consistent with reduced functional activity in the presence of shed hMSLN. Similar trends were observed in the pancreatic cancer model based on tumor bioluminescence signals and tumor weight on day 16 (Figure S6).

### 15B6 CAR-M treatment generates durable systemic protection against tumor rechallenge

To determine whether mice that achieved CR developed a long-term anti-tumor immune response, CR mice were rechallenged at a distant site with either hMSLN^+^ CT26M or hMSLN^−^ CT26 tumor cells. As shown in Figure 5D, native TPO-MSLN or BALB/c mice developed progressive subcutaneous tumors, whereas CR mice rejected both hMSLN^+^ and hMSLN^−^ tumors.

Together, these findings demonstrate that 15B6 CAR-Ms mediate robust anti-tumor activity *in vivo*, outperforming SS1 CAR-Ms and BMDM controls. Importantly, tumor regression following 15B6 CAR-M treatment was associated with durable systemic protection against subsequent tumor challenge, consistent with the development of a broader host anti-tumor immune response.

## Discussion

In this study, our data support antigen shedding as one important barrier that can limit CAR-M’s activity against solid tumors. Using MSLN as a model target, we show that both shed and membrane-retained MSLN epitopes are present on the surface of the tumor cells (Figure S7) and that MSLN shedding is measurable both *in vitro*^9^ and *in vivo* (Figure S8). We further demonstrate that soluble shed hMSLN markedly impairs the anti-tumor activity of SS1 CAR-Ms, which recognize an N-terminal epitope contained within the shed extracellular domain, whereas 15B6 CAR-Ms, which bind a membrane-proximal non-shed epitope, retain robust cytotoxic activity under the same conditions. In immunocompetent hMSLN-tolerant models, targeting the membrane-proximal non-shed epitope resulted in substantially improved anti-tumor efficacy, including durable complete responses and protection against tumor rechallenge. Together, these findings establish shed antigen interference as a functionally important mechanism limiting CAR-M efficacy and show that rational epitope selection can overcome this barrier. Notably, epitope-dependent differences in immune synapse geometry may also contribute to the enhanced activity of membrane-proximal CAR-Ms.^21^^,^^22^ Thus, the benefit of this epitope-selective targeting strategy may reflect both reduced interference by shed MSLN and favorable membrane-proximal binding geometry.

In our earlier studies, we demonstrated that soluble MSLN can interfere with CAR-T receptor engagement and contribute to therapeutic resistance.^9^ Unlike T cells, which can initiate activation through transient receptor signaling, macrophages rely on sustained target engagement and iterative receptor triggering to execute phagocytosis and cytotoxic clearance.^15^ In this context, soluble antigen may act not only as a competitive binding decoy^9^^,^^10^ but also as a factor that limits the sustained signaling required for effective macrophage effector function. Consistent with this, our data show that physiologically shed hMSLN strongly inhibited SS1 CAR-Ms but had a negligible effect on 15B6 CAR-Ms, whereas full-length hMSLN containing the 15B6 epitope inhibited both constructs (Figure 4), indicating that susceptibility to soluble antigen interference is epitope dependent. These findings may have broader implications, as many CAR-M targets under investigation, including HER2, EGFR, and GD2, are also actively shed from the cell surface.^23^^,^^24^^,^^25^ By showing that a membrane-proximal, non-shed epitope preserves CAR-M activity in the presence of shed antigen, our study identifies epitope selection as an important design principle for macrophage engineering, and targets that undergo extensive proteolytic shedding but retain an accessible membrane-proximal epitope are expected to derive the greatest benefit from this shedding-resistant CAR-M design.

An important strength of this study is the use of immunocompetent hMSLN-tolerant models, which enabled evaluation of CAR-M activity in the context of intact host immunity while avoiding rejection of hMSLN-expressing tumor cells as foreign. This is particularly important for macrophage-based therapies, as CAR-Ms not only mediate direct tumor cell killing through phagocytosis but also actively shape the broader immune response through antigen presentation^3^ and cytokine production.^26^ Prior studies have often relied either on immunodeficient mouse models,^4^^,^^16^ which allow engraftment of murine tumors engineered to express a human target antigen but fail to capture the full interaction between CAR-Ms and the host immune system, or on immunocompetent models,^27^^,^^28^ in which responses against the foreign antigen itself may confound interpretation of efficacy and lead to overestimation of the therapeutic effect. By contrast, the hMSLN-tolerant models used here resolved this limitation by allowing evaluation of CAR-M function in the context of intact host immunity without confounding foreign antigen responses. In these settings, 15B6 CAR-Ms showed substantially greater anti-tumor activity than in NSG mice and induced CR in 35% of tumor-bearing animals (Figure 5), highlighting the value of an immunocompetent, antigen-tolerant platform for assessing CAR-M function *in vivo*.

Importantly, these complete responses were accompanied by rejection of rechallenge with both hMSLN^+^ and hMSLN^−^ CT26 tumor cells. CT26M cells were generated from parental hMSLN^−^ CT26 cells, so these two lines share most of their tumor-associated antigens but differ in hMSLN expression. Because this protection extended beyond the original CAR target, it is unlikely to be explained solely by persistence of infused 15B6 CAR-Ms or by a narrowly hMSLN-restricted effector mechanism. Previous studies have demonstrated that CAR-Ms can mediate antigen cross-presentation to CD8 T cells following tumor cell phagocytosis.^3^^,^^29^ Therefore, one possible mechanism is that CAR-dependent phagocytosis and killing of hMSLN^+^ CT26M cells release a broad repertoire of tumor-derived antigens. These antigens may be processed and cross-presented by the infused CAR-Ms and/or endogenous APCs, thereby promoting epitope spreading and activating adaptive anti-tumor immunity against additional, MSLN-independent tumor antigens. Such immunity may help sustain the efficacy of single-antigen therapies in solid tumors, where antigen heterogeneity and antigen-loss escape commonly limit durable responses. In this regard, the ability of 15B6 CAR-Ms to preserve direct anti-tumor activity under shed-antigen conditions while also promoting broader host immune protection may represent a key advantage over conventional CAR-M designs that remain vulnerable to soluble antigen blockade. However, although our findings are consistent with cross-presentation and epitope spreading, the underlying immune mechanisms were not directly examined in the present study.

In addition, our study establishes a practical approach for *ex vivo* generation of murine CAR-Ms. RetroNectin-mediated lentiviral transduction enabled efficient gene delivery into bone marrow-derived myeloid precursor cells, which subsequently differentiated into macrophages and supported continued expansion, whereas polybrene-based transduction, despite comparable initial transduction rates, failed to support sustained cell growth (Figure S9). This difference may reflect better preservation of precursor viability under RetroNectin-based conditions during early myeloid differentiation, consistent with prior reports that polybrene is less compatible with primary macrophages.^30^^,^^31^^,^^32^ Although not a primary focus of the study, this finding highlights the importance of optimizing engineering conditions during early myeloid differentiation to generate functional murine CAR-M products. However, given the differences between murine and human macrophages, further studies using human cell platforms will be required to determine the translational applicability of this engineering strategy.

This study has several limitations. First, although our rechallenge data provide strong evidence that 15B6 CAR-M therapy can elicit broader host anti-tumor immunity, the precise pathways linking initial CAR-M-mediated tumor clearance to durable immune protection remain to be fully resolved. Tumor antigen release, uptake, and presentation are likely to contribute, but we did not directly test whether infused CAR-Ms drive this process through cross-presentation *in vivo*. Future studies combining immune cell depletion, adoptive transfer, and tumor immune profiling should help define the relative contributions of endogenous CD8^+^ T cells, CD4^+^ T cells, NK cells, and host myeloid populations to tumor control and long-term protection. Second, although the principle was validated across multiple hMSLN^+^ tumor models, its broader applicability to other shed tumor antigens remains to be established. Future studies will be needed to determine whether this shedding-resistant design strategy can be extended to other proteolytically shed tumor antigens.

In summary, our study identifies shed antigen interference as a key mechanism limiting CAR-M efficacy in solid tumors and demonstrates that targeting a membrane-proximal, non-shed epitope of MSLN can overcome this barrier. These findings provide a mechanistic explanation for the limited activity of conventional MSLN-directed CAR-Ms in the presence of soluble antigen and establish epitope selection as a central design parameter for macrophage-based cell therapy. More broadly, these results suggest that overcoming antigen shedding may be critical for realizing the full therapeutic potential of CAR-Ms against solid malignancies and support further development of shedding-resistant CAR strategies to improve both direct tumor clearance and durable systemic anti-tumor immunity.

## Materials and methods

### Cell culture

PDA223481 cells (described as KPCdelMsln in the original paper) were isolated from pancreatic ductal adenocarcinoma (PDAC) tumors in *Kras*^G12D/+^ *Trp53* ^R172H/+^:p-48-Cre (KPC):*Msln*^−/−^ mice as described earlier,^20^ and they are pancreatic tumor cells that do not express mouse Msln and hMSLN. H06 cells were PDA223481 cells that were transduced to express orthologous hMSLN protein using lentiviral transduction as described earlier.^20^ KPC911 cells were isolated from tumor tissue in transgenic mice that spontaneously developed PDAC that expresses hMSLN (by crossing de-floxed Msl mice^20^ with the KPC mice). AB1 was a mouse mesothelioma cell line purchased from Sigma-Aldrich. AB1MV cells were AB1 cells transduced with a recombinant lentivirus engineered to express hMSLN (pLenti-PGK-puro-hMSLN plasmid vector) to stably express hMSLN (593V) on the cell membrane. CT26 cells were murine colorectal tumor cells purchased from ATCC. CT26M was a high-expression clone stably expressing hMSLN generated and selected from the parental CT26 murine colon carcinoma cell line.^33^ The Luc-expressing tumor cells were produced for *in vitro* cytotoxicity assays and *in vivo* tumor experiments. All cells were cultured in DMEM supplemented with 10% fetal bovine serum (FBS) and 1% penicillin-streptomycin at 37°C with 5% CO_2_. Cell lines were regularly tested for mycoplasma contamination.

### Reagent used by flow cytometry

UltraComp eBeads (01-2222-42) and anti-mouse CD16/32 (14-0161-86) were purchased from Invitrogen. PE-anti-mouse/human CD11b (101207), PE/cy7-anti-mouse F4/80 (123114), PE-anti-mouse CD80 (104707), BV605-anti-mouse CD86 (105037), and APC-anti-mouse CD206 (141708) were purchased from BioLegend. Mor009-AF647 and 15B6-AF647 were conjugated by using anti-hMSLN antibody (clone Mor009 or 15B6) with the protein labeling kit (Thermo Fisher Scientific, Cat: A20186). Fc blocking by anti-mouse CD16/32 was performed when necessary.

### Generation and expansion of CAR-Ms

Plasmid DNA encoding CAR constructs comprising different murine scFvs (15B6, SS1, and FMC63), followed by the hinge and transmembrane regions of murine CD8, a murine intracellular co-stimulation domains (41BB, CD86, and TLR4), a murine CD3ζ, a self-cleaving T2A sequence, and GFP as a surrogate marker of CAR expression, was packaged into lentivirus (pWPT backbone) by co-transfecting with envelope vector (pMD2.G) and packaging vector (psPAX2) into HEK293T cells using Calfectin (SignaGen) as described previously.^34^ The sequences of the targeting domains used in this study are provided in Table S1.

Bone marrow cells were isolated from mice with either a C57BL/6 or BALB/c background on day 1 as previously described.^35^ The cells were then stimulated with 25 ng/mL M-CSF (PeproTech) for 72 h. On day 4, the unattached cells were washed away; the remaining cells were myeloid precursor cells and were detached with Accutase (Innovative Cell Technologies). The lentiviral transduction was performed (MOI = 10) on a RetroNectin pre-coated untreated cell culture dish (Takara), and the transduction efficiency was tested on day 7. After that, the transduced cells were passaged on non-treated dishes/flasks and maintained until they reached over 85% confluence before being collected for *in vitro* or *in vivo* experiments. We used BMDMs within five generations, as their proliferation efficiency declines significantly thereafter.

### MSLN-binding assay

15B6 CAR-Ms, SS1 CAR-Ms, or FMC63 CAR-Ms were pre-incubated with full-length hMSLN (hMSLN 296-599-His) on ice for 30 min, then washed with PBS. After the wash, 15B6 CAR-Ms and FMC63 CAR-Ms were incubated with AF647-labeled antibody that binds to the N terminus of hMSLN (Mor009), while SS1 CAR-Ms were incubated with AF647-labeled 15B6 antibody. The percentage of binding was calculated as 100% × GFP^+^ AF647^+^ cells/GFP^+^ cells as measured by flow cytometry. The binding activity of each construct was verified using BMDMs independently derived from at least two mice.

### Phagocytosis assay

Tumor cells of H06, KPC911, CT26M, and PDA223481 were live labeled with CellTracker Far Red Dye (Thermo Fisher Scientific) and co-cultured with CAR-Ms or BMDMs in a 96-well low-adherent plate at a 1:1 ratio. After 4 h, all cells were lifted by Accutase and analyzed by flow cytometry.

Two independent experiments were performed. The percentage of phagocytosis was calculated as the FarRed^+^ population within the gated macrophage population. As indicated in the flow gate, %Phagocytosis = (M^+^ FarRed^+^ cells/M^+^ cells) × 100%. Here, M represents effector cells (marked by either GFP, F4/80, or CD11b), depending on the experiment.

### Luminescent-based cytotoxicity assay

An *in vitro* cell-killing assay was performed as described before.^9^ Effector CAR-Ms were co-cultured with Luc^+^ target cells at different E/T ratios in a 96-well plate for 24, 48, or 72 h. Full-length hMSLN (296-599-His) or shed hMSLN (296-585-His) was added to test their influence. At the end of the co-culture period, cells were lysed for 15 min in lysis buffer. The Luc activity in the lysates was measured using the Luc assay system (Promega), and results were analyzed as the percentage of killing: % killing = 100 – 100 × (relative light units [RLUs] from wells with effector and target cells)/(average RLUs from wells with target cells). All experiments were independently repeated with two biological replicates.

### Macrophage phenotyping by flow cytometry

15B6 CAR-Ms or BMDMs were co-cultured with H06 or PDA223481 cells at E/T ratio = 1 on 96 well plates. 48 h later, macrophages (15B6 CAR-Ms: GFP^+^ F4/80^+^, BMDMs: F4/80^+^) were analyzed by flow cytometry. Expression of CD80 and CD86 was measured as markers commonly associated with M1-like macrophages, whereas CD206 was measured as a marker commonly associated with M2-like macrophages. Macrophages stimulated with 50 ng/mL IFN-gamma (PeproTech) and 20 ng/mL LPS (MilliporeSigma) were set as positive controls for the M1-like macrophage phenotype. Macrophages that were stimulated with 20 ng/mL IL-4 (PeproTech) were set as positive controls for the M2-like phenotype. Two independent experiments were performed.

### Mouse experiments

All mouse experiments were approved by the NCI Animal Care and Use Committee (protocol number: LMB-014). Male and female mice at 8–16 weeks were included in the study, with male mice labeled “M” in the figures. Mice were randomly assigned to experimental groups. The BALB/c TPO-MSLN transgenic mouse expressing hMSLN in the thyroid under the control of the TPO promoter was generated in our lab as described previously.^19^ The C57BL/6 hMSLN (described as Msl mouse in the original paper) transgenic mouse was a transgenic mouse expressing hMSLN where mouse Msln should be expressed.^20^ Both TPO-MSLN mice and hMSLN mice do not reject genetically modified mouse tumor cells expressing hMSLN. NSG mice were purchased from Jackson Lab.

For the colorectal tumor model, 0.5 million CT26M Luc^+^ tumor cells were injected (intraperitoneally [i.p.]) into TPO-MSLN mice or NSG mice (8–16 weeks) on day 1. Dosage and treatment schedule of 15B6 CAR-Ms, SS1 CAR-Ms, BMDMs, or Veh (fresh DMEM) are listed in each figure legend. Mice were euthanized if their weight loss exceeded 15% or they looked moribund. All mice were followed for at least 45 days. The survival of mice in each treatment group was recorded. Mice were imaged using Xenogen IVIS Lumina (PerkinElmer) after 10 min of 1.5 mg d-luciferin (PerkinElmer) injection (i.p.). All images were analyzed by Living Image software (PerkinElmer). The luminescent intensity (LI) of photons emitted from tumors in each mouse was quantified.

For the pancreatic cancer model, 0.5 million H06 Luc^+^ tumor cells were injected (i.p.) into hMSLN mice (8–16 weeks) on day 1. Two hours post-tumor injection, 1 × 10^7^ 15B6 CAR-Ms (*n* = 7), 1 × 10^7^ SS1 CAR-Ms (*n* = 3), or Veh (DMEM, *n* = 9) were injected (i.p.). On day 16, 6 mice in the Veh group, 4 mice in the 15B6 CAR-M group, and 3 mice in the SS1 CAR-M group were euthanized, and the tumors in the abdominal cavity were weighed. Three mice in the Veh group and 3 mice in the 15B6 CAR-M group were left to check whether they reached CR. Mice were imaged using Xenogen IVIS Lumina (PerkinElmer) after 10 min of 1.5 mg d-luciferin (PerkinElmer) injection (i.p.). All images were analyzed by Living Image Software (PerkinElmer). The LI of photons emitted from tumors in each mouse was quantified.

In rechallenge assays, the mice with CR were rechallenged with either 2 × 10^6^ CT26M or CT26 cells given subcutaneously 1.5 months after initial cell inoculation. The size of the tumors was measured 2–3 times a week, and mice were euthanized if tumor size exceeded 400 mm^3^ or if the mice looked moribund. Tumors in the abdominal cavity of each mouse in the rechallenge assay were checked at each experimental endpoint (euthanization day or last day in the record). Mice that died with both subcutaneous tumor and tumor in the abdominal cavity were not considered as CR from the treatment. Mice rejecting the subcutaneous tumor were considered to have developed anti-tumor immunity.

### Statistical analysis

We used Prism 10 for all statistical calculations and graph fitting. Quantitative results were expressed as the mean ± standard deviation (SD). Shapiro-Wilk and Levene tests were performed to check the normality and homogeneity of variance, respectively. The *t* test and analysis of variance (ANOVA) were used for group comparisons of two or more two groups, respectively. To compare the survival of groups, we used the Kaplan-Meier test. The Mann-Whitney U test was used for the nonparametric analysis. *p* < 0.05 was considered statistically significant.

## Data and code availability

All data relevant to the study are included in the article or uploaded as supplemental information.

## Acknowledgments

We thank Serguei Kozlov at the Center for Advanced Preclinical Research for providing hMSLN transgenic mice and KPC911 cells, Christine Alewine at the Laboratory of Molecular Biology for providing the H06 and PDA223481 cells, and Swati Priya at the Laboratory of Molecular Biology for helping to prepare the manuscript. The authors would also like to thank the CCR Genomic Core and Flow Core (NCI) for the use of their facilities. This research was supported (in part) by the Intramural Research Program of the 10.13039/100000002National Institutes of Health (NIH) and Evotec. The contributions of the NIH authors were made as part of their official duties as NIH federal employees, are in compliance with agency policy requirements, and are considered works of the United States government. However, the findings and conclusions presented in this paper are those of the authors and do not necessarily reflect the views of the NIH or the US Department of Health and Human Services.

## Author contributions

W.L. and I.P. designed the research; W.L., X.L., S.K., Q.Z., M.O., and N.W. performed the research; X.L. and M.O. contributed new reagents/analytic tools; W.L. analyzed the data; W.L. prepared the figures; and W.L. and I.P. wrote the paper.

## Declaration of interests

I.P., M.H., X.L., and M.O. are inventors of patents on the 15B6 antibody and have assigned all rights to the NIH.

## Declaration of generative AI and AI-assisted technologies in the manuscript preparation process

During the preparation of this work, the author used ChatGPT in order to improve the grammar, phrasing, and clarity of the manuscript. After using this tool/service, the authors reviewed and edited the content as needed and take full responsibility for the content of the published article.

## Supplemental information

Document S1. Figures S1–S9 and Table S1

Document S2. Article plus supplemental information
