# Supplementary material for: Epitope selection overcomes antigen shedding to enable effective CAR-M therapy in solid tumors
Source: Mol Ther Oncol. 2026 Sep 30;34(4):201354. doi: 10.1016/j.omton.2026.201354 (PMC13692139; doi:10.1016/j.omton.2026.201354)
Supplement: Supplementary file 1 [file mmc1.pdf]

OMTON, Volume *34*

Supplemental information

Epitope selection overcomes antigen shedding to enable effective CAR-M therapy in solid tumors

Wenlong Liu, Xiufen Liu, Seraphim Kozlov, Qi Zhou, Masanori Onda, Nadja Wagner, and Ira Pastan

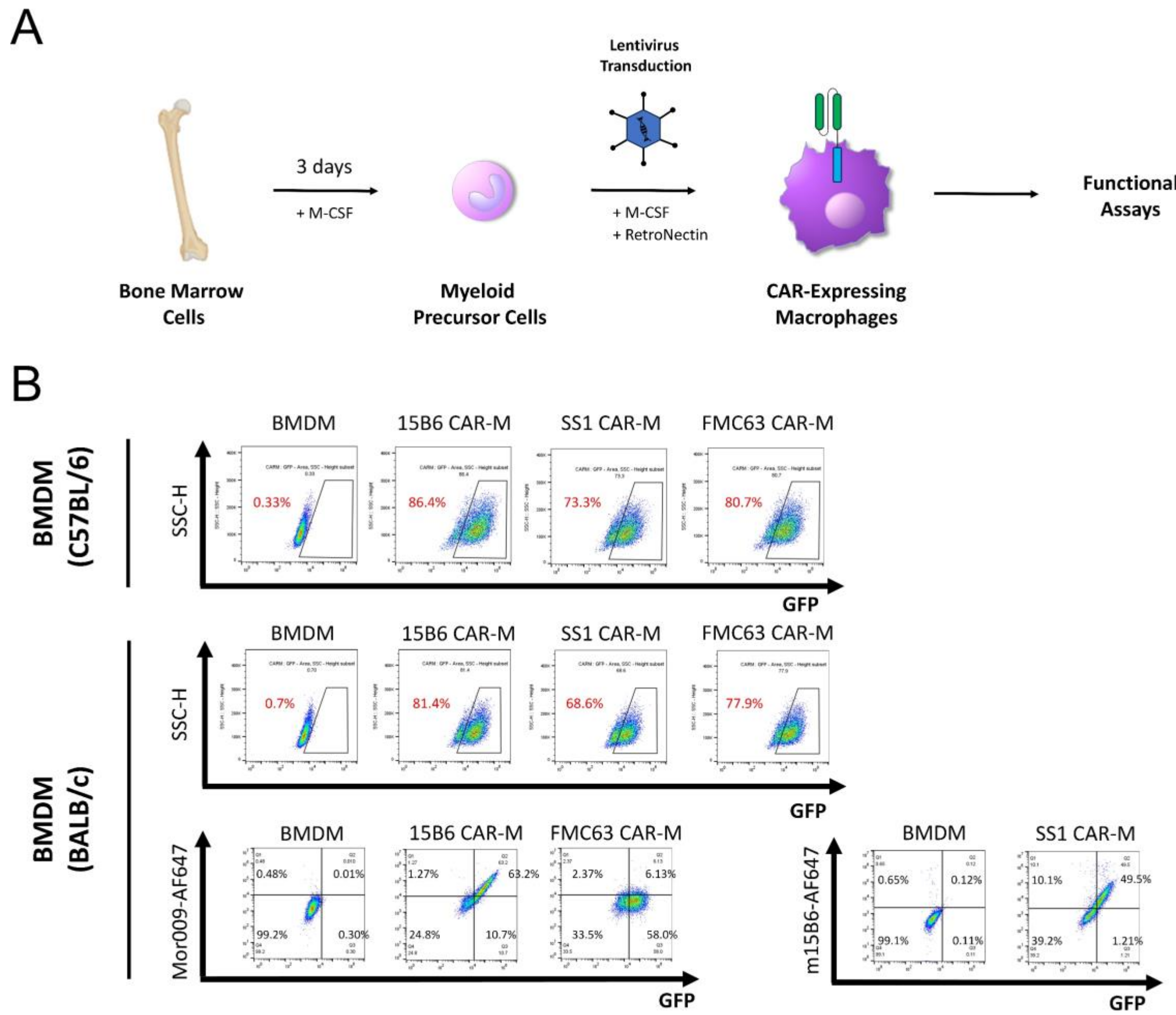

Figure S1. Transduction and MSLN-binding activity of CAR-M derived from BMDMs of different mouse strains. A. Schematic representing the CAR-M manufacturing process. B. Bone marrow cells of mice with either C57BL/6 background (hMSLN mice) or BALB/c background (TPO-MSLN) were transduced to express CARs. The transduction rates were measured by % GFP$^+$ cells. Successfully transduced CAR-M were GFP$^+$. Cells were then incubated with hMSLN (295-599-His) on ice, following by anti-hMSLN staining. Transduced cells that bind to hMSLN were AF647$^+$ GFP$^+$. %Binding = (% AF647$^+$ GFP$^+$ cells / % GFP$^+$ cells) x 100%.

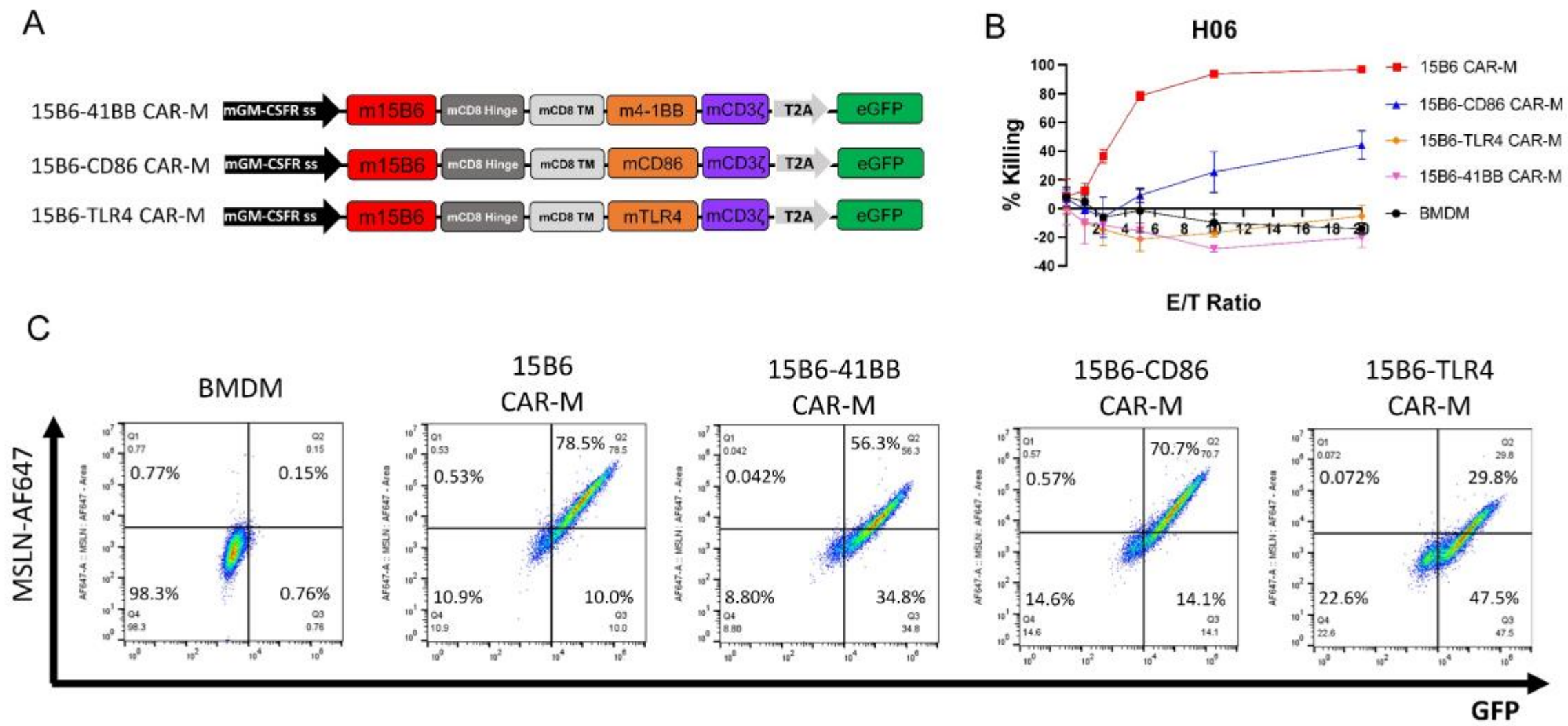

Figure S2. **Adding a co-stimulatory domain does not increase 15B6 CAR-M's cytotoxicity.** A. CAR constructs used in macrophages. B. Cytotoxicity of 15B6 CAR-M, 15B6-41BB CAR-M, 15B6-CD86 CAR-M, and 15B6-TLR4 CAR-M were measured by co-culturing CAR-M or BMDM with Luc^{+} H06 cells on 96 well plates at different E/T ratios for 48 hours. C. CAR-M were incubated with MSLN (295-599-His) on ice, followed by anti-MSLN AF647 staining. Transduced cells that bind to MSLN were $AF647^{+}$ GFP^{+}. %Binding = (% $AF647^{+}$ GFP^{+} cells / % GFP^{+} cells) x 100%.

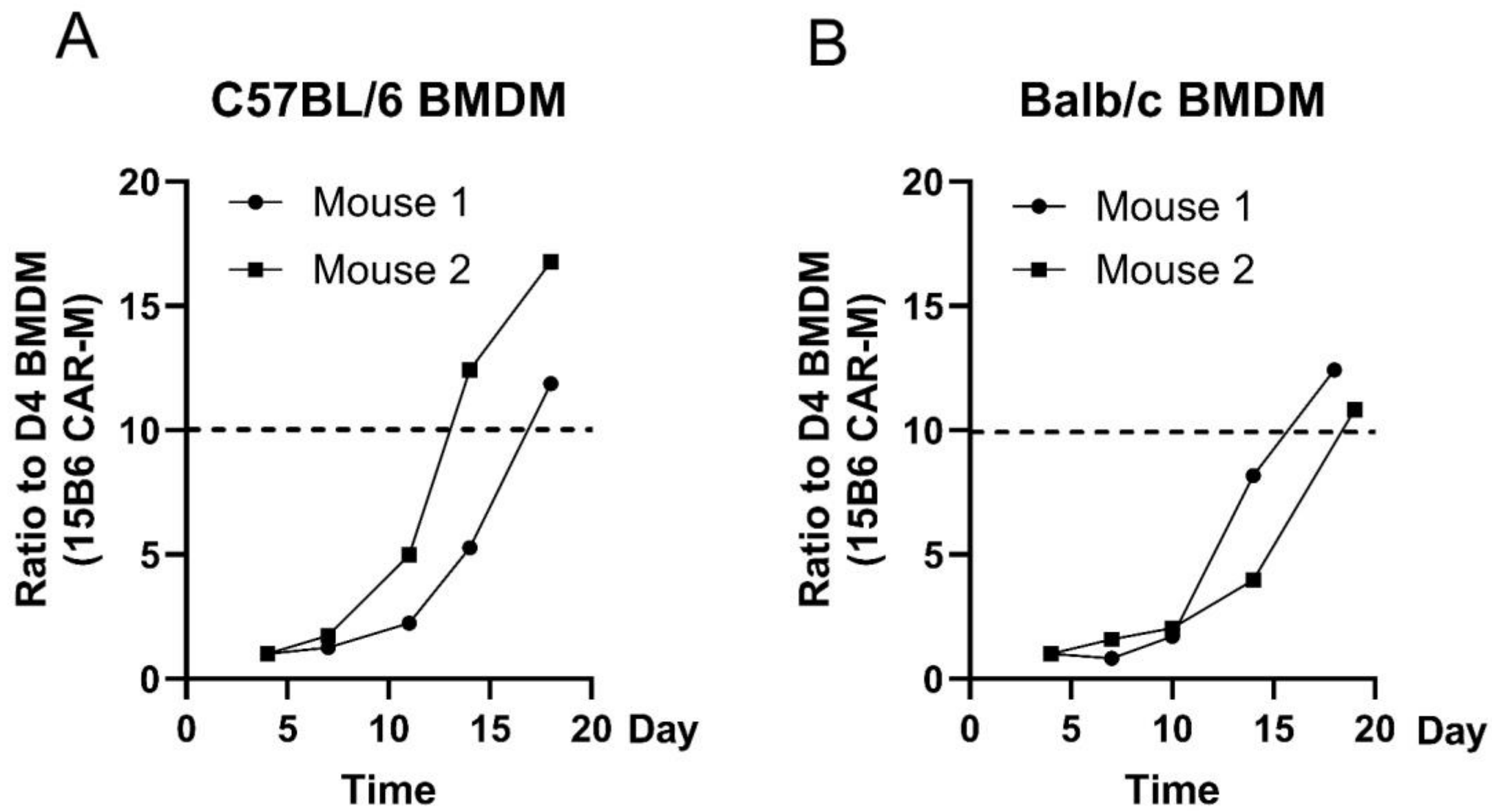

Figure S3. **CAR-M generated via RetroNectin-assisted transduction retain their expansion capability.** Proliferation curve of bone marrow cells isolated from either mouse with C57BL/6 background (A) or Balb/c background (B) and transduced with 15B6 CAR-M lentivirus using RetroNectin.

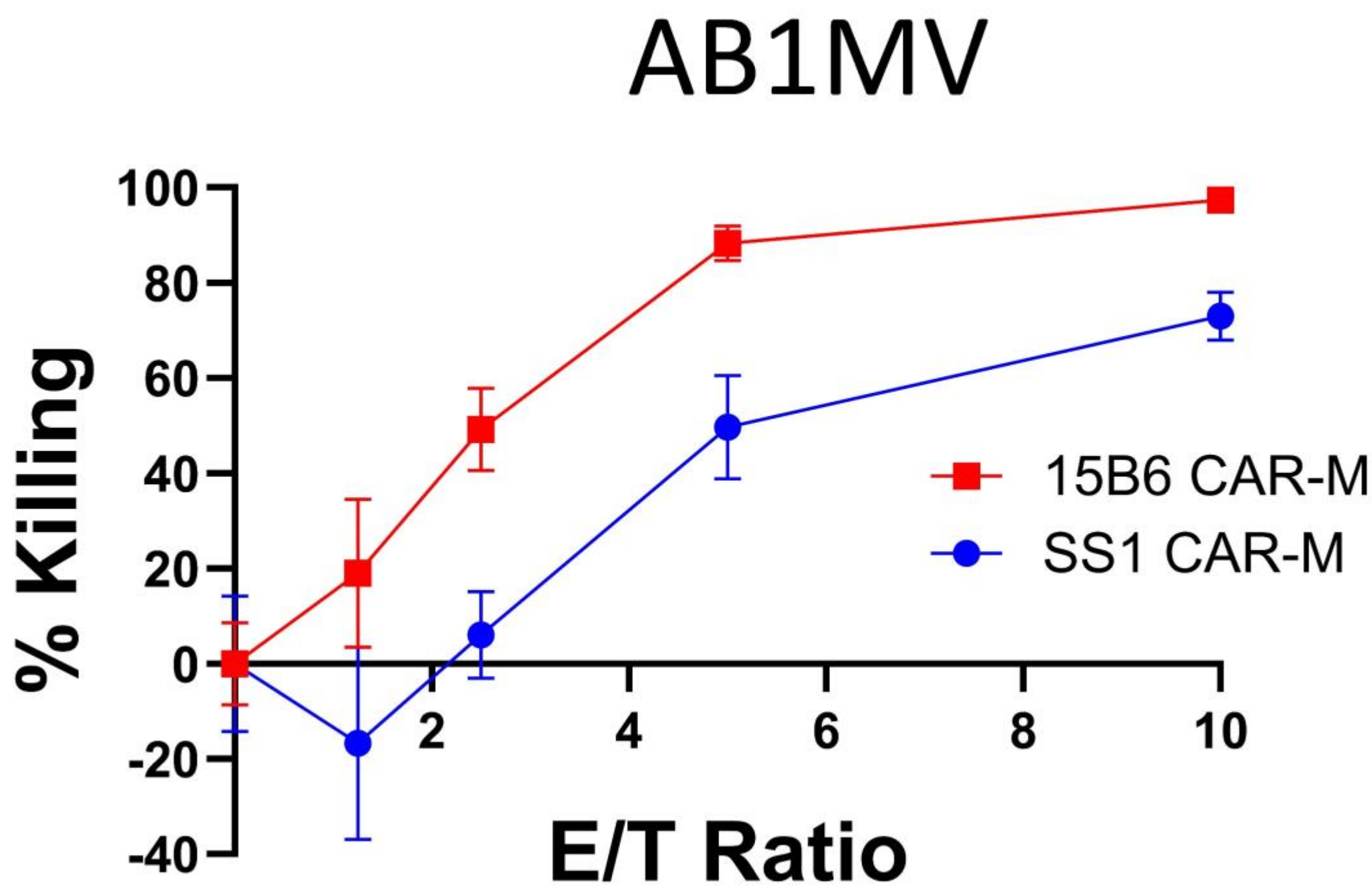

Figure S4. 15B6 CAR-M show better killing activity than SS1 CAR-M on AB1MV cells. Co-culture of 15B6 CAR-M or SS1 CAR-M with Luc⁺ AB1MV cells on 96 well plates at different E/T ratios for 48 hours.

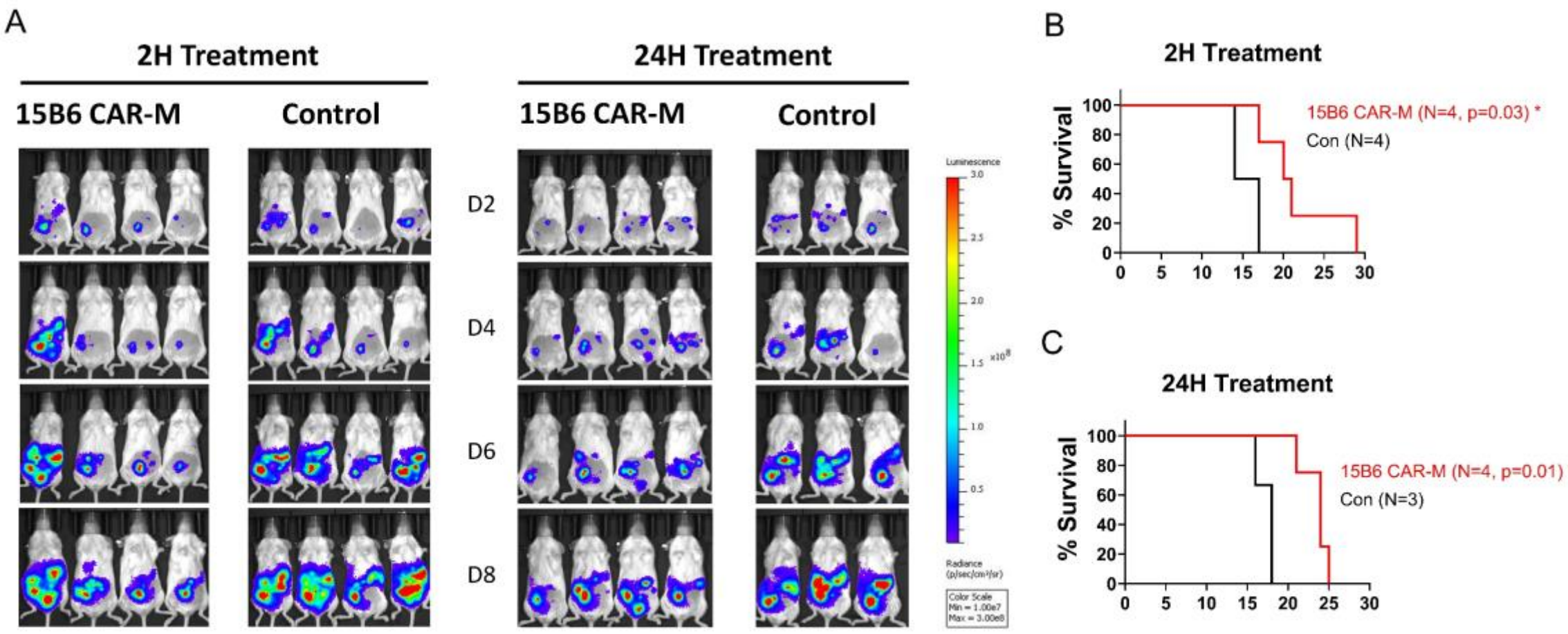

Figure S5. 15B6 CAR-M significantly prolonged survival of NSG mice bearing colorectal cancer. NSG mice were inoculated (i.p.) with 0.5E6 CT26M cells on day one and treated (i.p.) with 1E7 15B6 CAR-M or DMEM either 2H or 24H post-tumor inoculation. Mice were IVIS imaged (A) and the survival was calculated (B-C).

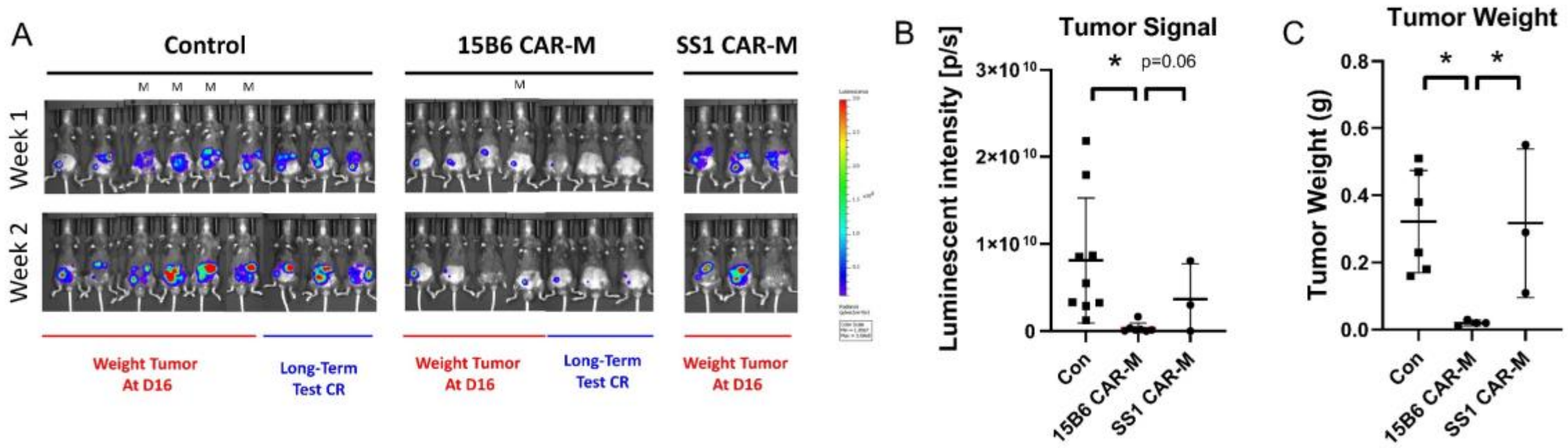

Figure S6. 15B6 CAR-M show superior anti-tumor activity compared to SS1 CAR-M in the treatment of pancreatic tumor in hMSLN mice. hMSLN mice were inoculated (i.p.) with 0.5E6 H06 cells on day one and treated (i.p.) with 1E7 15B6 CAR-M, SS1 CAR-M or DMEM 2 h post-tumor inoculation. Mice were IVIS imaged (A). Luminescent signal intensity (B) and tumor weight (C) at day 16 were measured. (A) 3 mice in Veh control group and 3 mice in 15B6 CAR-M group were followed further to determine whether CR were achieved.

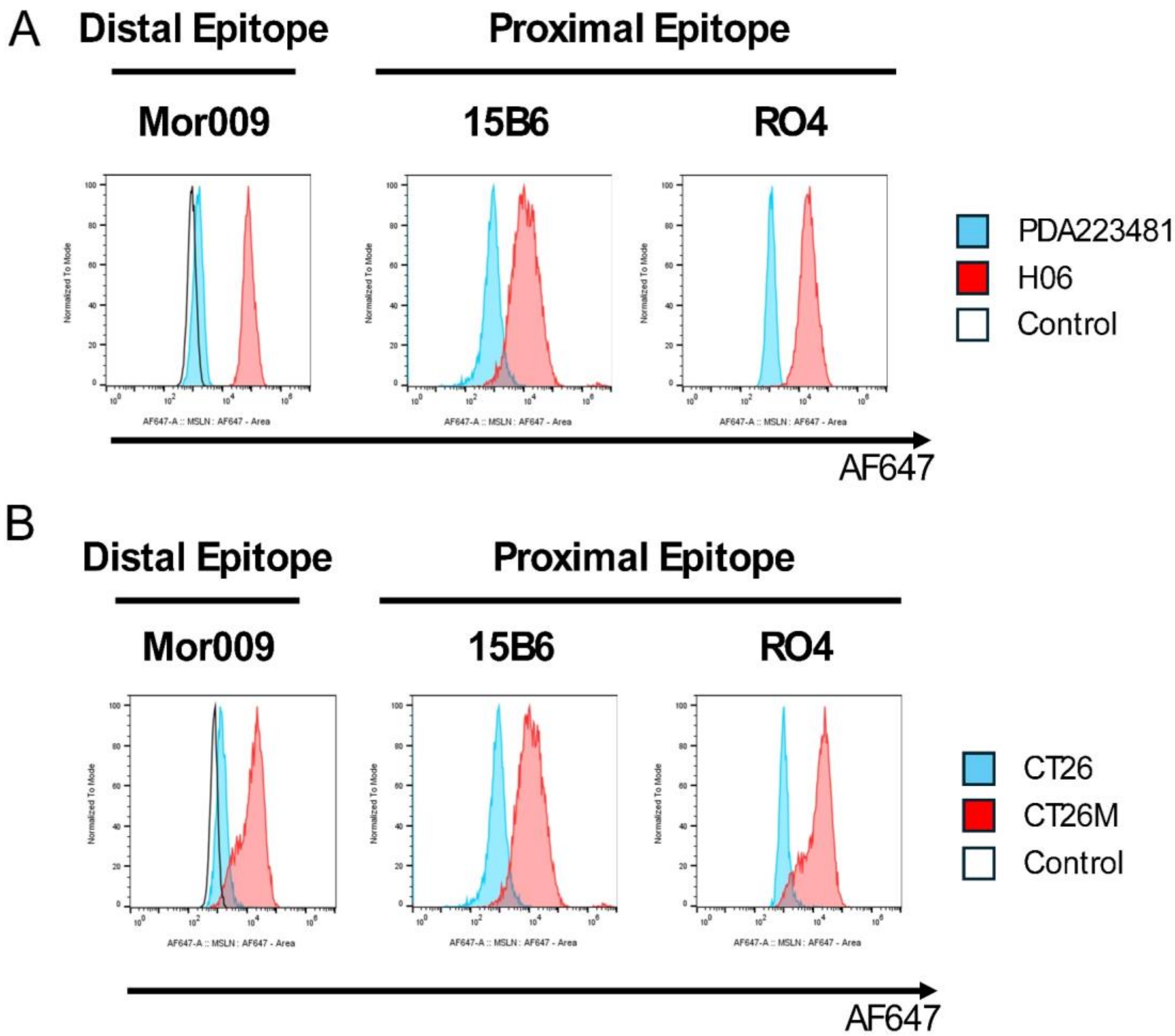

Figure S7. MSLN surface staining of target cells. (A) H06 and PDA223481 cells and (B) CT26M and CT26 cells were individually analyzed by flow cytometry following staining with the antibodies recognizing the membrane-distal region of MSLN (Mor009) and the membrane-proximal region of MSLN (15B6 and RO4).

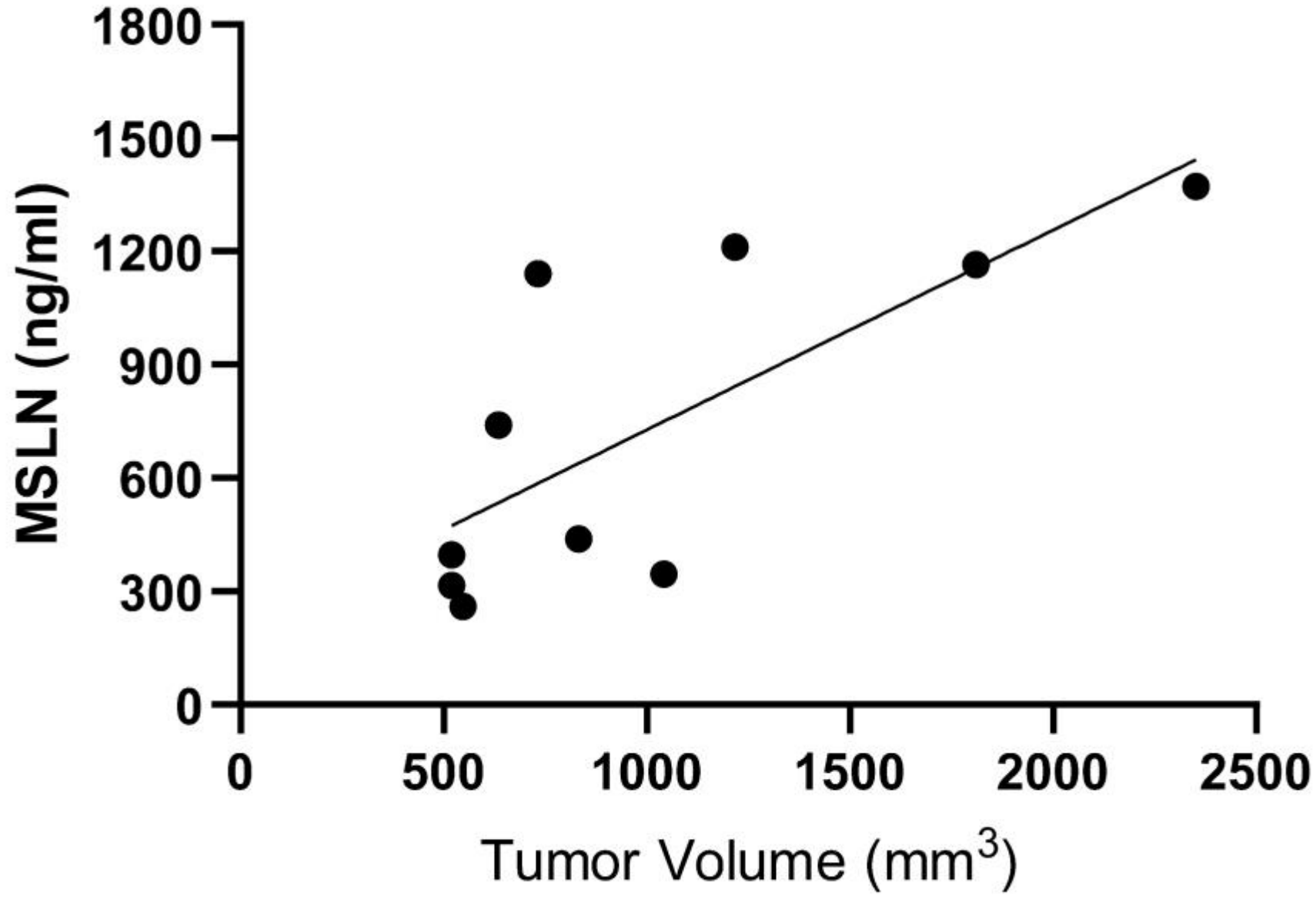

Figure S8. Correlation between tumor volume and shed mesothelin levels in the serum of CT26M tumor-bearing mice. Serum shed MSLN levels were positively correlated with tumor volume (R = 0.74; P = 0.0138).

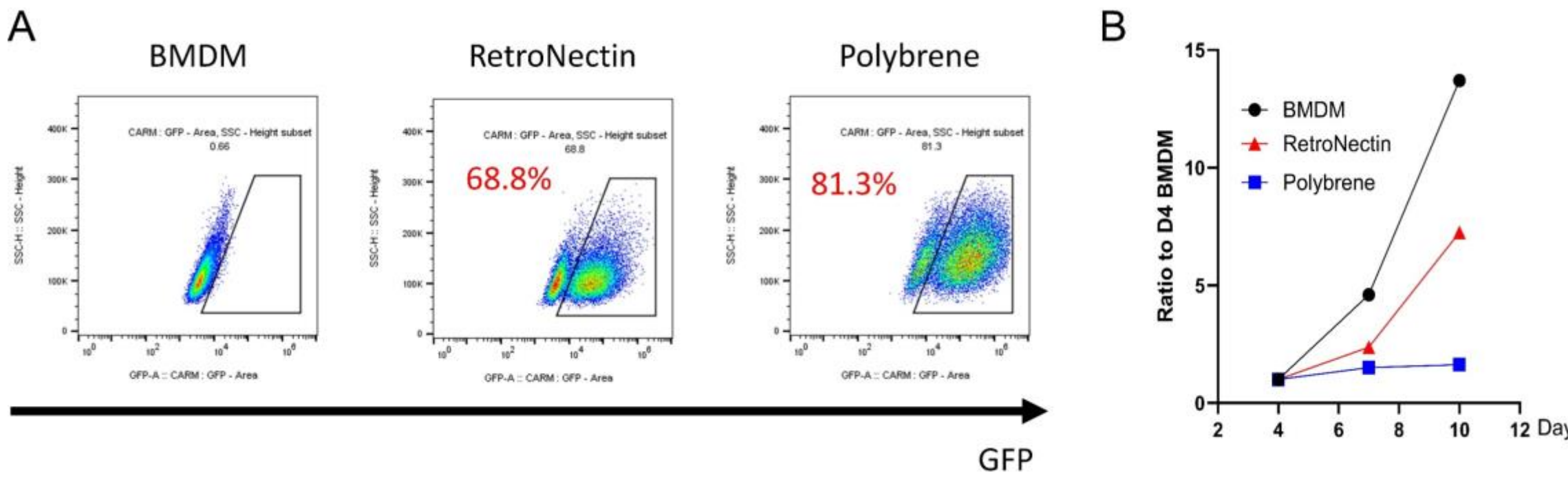

Figure S9. Transduction with polybrene inhibits bone marrow cell expansion. A. Bone marrow cells of mice with BALB/c background (TPO-MSLN) were transduced on day 4 in the presence of either polybrene or RetroNectin. The transduction rates were measured by % GFP⁺ cells at D10. B. Proliferation curves of the transduced cells.

Table S1. Sequence of the targeting domains in each CAR-M construct

Construct	Sequence
15B6 scFv	QAVVTQESALTTSPGETVTLTCRSSTGAVTTGNYPNWVQEKPDHLFTGLIAGTNNRAP<br>GVPARFSGSLIGDKAALTITGAQTEDEAIYFCALWFSSHWVFGGGTKLTVLGGGGSGG<br>GGSGGGGSEVQLQQSGPVLVKPGASVKISCKASGYSFTGYYMHWVRQSLVKRLEWIG<br>RINPYTGVPSYKHNFKDKASLTVDKSSSTAYMELHSLTSEDSAVY<br>YCARELGGYWGQGTTLTVSS
SS1 scFv	QVQLQQSGPELEKPGASVKISCKASGYSFTGYTMNWVKQSHGKSLEWIGLITPYNGAS<br>SYNQKFRGKATLTVDKSSSTAYMDLLSLTSEDSAVYFCARGGYDGRGFDYWGQGTTVT<br>VSSGGGGSGGGGSGGGGSDIELTQSPAIMSASPGEKVTMTCSASSSVSYMHWYQQK<br>SGTSPKRWIYDTSKLASGVPGRFSGSGSGNSYSLTISSVEAEDDATYYCQQWSKHPLTF<br>GAGTKLEIK
FMC63 scFv	DIQMTQTTSSLSASLGDRVTISCRASQDISKYLNWYQQKPDGTVKLLIYHTSRLHSGVP<br>SRFSGSGSGTDYSLTISNLEQEDIATYFCQQGNTLPYTFGGGTKLEITGGGGSGGGGSG<br>GGGSEVKLQESGPGLVAPSQSLSVTCTVSGVSLPDYGVSWIRQPPRKGLEWLGVIWGS<br>ETTYYNSALKSRLTIIKDNSKSQVFLKMNSLQTDDTAIYYCAKHYYYGGSYAMDYWGQG<br>TSVTVSS
